# Mesenchymal stromal cells attenuate sevoflurane-induced apoptosis in human neuroglioma H4 cells

**DOI:** 10.1186/s12871-018-0553-1

**Published:** 2018-07-18

**Authors:** Yanyong Cheng, Yunfeng Jiang, Lei Zhang, Jiayi Wang, Dongdong Chai, Rong Hu, Chunzhu Li, Yu Sun, Hong Jiang

**Affiliations:** Department of Anesthesiology, Shanghai Ninth People’s Hospital Affiliated to Shanghai Jiao Tong University School of Medicine, Center for Specialty Strategy Research of Shanghai Jiao Tong University China Hospital Development Institute, 639 Zhizaoju Road, Shanghai, 200011 China

**Keywords:** Sevoflurane, Mesenchymal stem cells, Reactive oxygen species, Apoptosis, mitochondrial dysfunction

## Abstract

**Background:**

Inhalation of sevoflurane can induce neuronal apoptosis, cognitive impairment and abnormal behaviors. Bone marrow mesenchymal stem cells (MSCs) can secret neurotrophic factors and cytokines to protect from oxidative stress-related neuronal apoptosis. However, whether MSCs can protect from sevoflurane-induced neuronal apoptosis and the potential mechanisms are unclear.

**Methods:**

A non-contact co-culture of MSCs with human neuroglioma H4 cells (H4 cells) was built. H4 cells were co-cultured with MSCs or without MSCs (control) for 24 h. The co-cultured H4 cells were exposed to 4% sevoflurane for 6 h. The levels of caspase-3, reactive oxygen species (ROS), adenosine triphosphate (ATP), and the release of cytochrome C were determined by Western blot and fluorescence assay.

**Results:**

Sevoflurane exposure significantly elevated the levels of cleaved caspase 3 and Bax in H4 cells. However, these phenomena were significantly offset by the co-culture with MSCs in H4 cells. Co-culture with MSCs before, but not after, sevoflurane exposure, significantly attenuated sevoflurane-induced ROS production in H4 cells. MSCs prevented sevoflurane-mediated release of cytochrome C from the mitochondria and production of ATP in H4 cells.

**Conclusions:**

Our study indicated that soluble factors secreted by MSCs attenuated the sevoflurane-induced oxidative stress and apoptosis of neuronal cells by preserving their mitochondrial function.

## Background

Sevoflurane is an inhaled anesthetic for both adult and pediatric anesthesia frequently used in clinical practice, and has characteristics of low blood:gas coefficient, rapid onset and recovery. However, sevoflurane exposure can induce severe neurological side effects, such as cognitive impairment and abnormal behaviors, like autism spectrum disorder [1–5]. Our previous studies have shown that such adverse effects of sevoflurane may be associated with the dysfunction of the LIMK1-signaling pathway and downregulation of circulating insulin-like growth factor 1 in developing and aged rats [6, 7]. Furthermore, sevoflurane exposure is prone to development of cognitive dysfunction in rodents and monkeys [3, 8, 9]. Similarly, a prospective randomized parallel-group study shows that inhalation of sevoflurane can promote the progression of amnestic mild cognitive impairment [10]. Moreover, sevoflurane exposure can change neurocognitive development and brain structure, leading to an increased risk of cognitive dysfunction in pediatric patients [2, 11–13]. However, the mechanisms underlying the neurotoxicity of sevoflurane against neuronal cells have not been clarified.

Sevoflurane can stimulate reactive oxygen species (ROS) production and induce neurodegeneration and synaptic loss, including mitochondria dysfunction [14, 15]. Indeed, antioxidant and inhibitors for nicotinamide adenine dinucleotide phosphate oxidase (NADPH) can protect against long-term memory impairment and neuronal apoptosis in rodents by reducing superoxide levels [16]. Hence, antioxidants and inhibition of ROS production can potentially inhibit sevoflurane-related neuronal toxicity.

Recent studies have demonstrated that mesenchymal stem cells (MSCs) can secrete neurotrophic factors, chemokines and cytokines, which have potent neuroprotective effects [17–20]. Infusion with MSCs improves ischemia reperfusion-induced neuronal injury in rodents [21, 22]. Previous clinical trials have demonstrated that MSC-based therapies are safe and effective for some diseases [23, 24]. Accordingly, we hypothesize that soluble factors secreted by MSCs can protect from sevoflurane-induced oxidative stress and neuronal apoptosis by preserving the mitochondrial function.

Glial cells regulate the synapse development and neuronal dysfunction [25], such as Alexander disease that manifests leukodystrophy and intellectual disability. Human neuroglioma H4 cells (H4 cells) share some similarities with glial cells in vitro and have been widely used for studies of neuronal impairment and apoptosis [26–28]. In this study, we employed a transwell-based co-culture system to determine the impact of soluble factors secreted by MSCs on sevoflurane exposure-induced ROS production, apoptosis and mitochondrial dysfunction in H4 cells in vitro.

## Methods

### Cell cultures

H4 cells were obtained from central laboratory of Shanghai Ninth People’s Hospital Affiliated to Shanghai Jiao Tong University School of Medicine and cultured in Dulbecco’s modified eagle medium (DMEM, 4.5 g/L glucose, Hyclone, USA) containing 10% fetal bovine serum (FBS, Gibco, USA), 100 Units/ml penicillin and 100 μg/ml streptomycin (Gibco, USA). Bone marrow-derived MSCs were isolated from newborn male Sprague-Dawley rats (SD rats) (6–7 days). Briefly, SD rats were sacrificed and their femurs were dissected, followed by flushing with DMEM (1 g/L glucose). The isolated MSCs were cultured onto 10-cm plastic dish and exchanged with fresh medium every three days. The third passage of MSCs was used for the co-culture system.

### Treatment with MSCs before sevoflurane exposure in H4 cells

The effect of pretreatment with MSCs on the production of ROS and apoptosis of H4 cells was determined by a non-contact co-culture system. MSCs (1 × 10^4^ cells/well, an optimal number determined by preliminary study) were cultured in DMEM (1 g/L glucose) in the upper chambers (6.5 mm inserter, 0.4 μm Polycarbonate Membrane, Costar, USA) while H4 cells (1 × 10^5^ cells/well) were cultured in DMEM (4.5 g/L glucose) in the bottom chambers at 37 °C in an incubator containing 21% O_2_ and 5% CO_2_ for 24 h. The upper chambers of the H4 cells without MSCs pretreatment group were filled with DMEM (1 g/L glucose) only and served as the control. After the co-culture with MSCs, the upper chambers were removed and the culture medium was changed with new medium. The H4 cells in the bottom chambers were exposed to 4% Sevoflurane or in an incubator for 6 h. The levels of intracellular ROS and apoptosis in H4 cells were detected at 0 and 24 h post sevoflurane exposure. Furthermore, the levels of ROS and apoptosis of H4 cells co-cultured with or without MSC pretreatment were also detected.

**Fig. 1:**
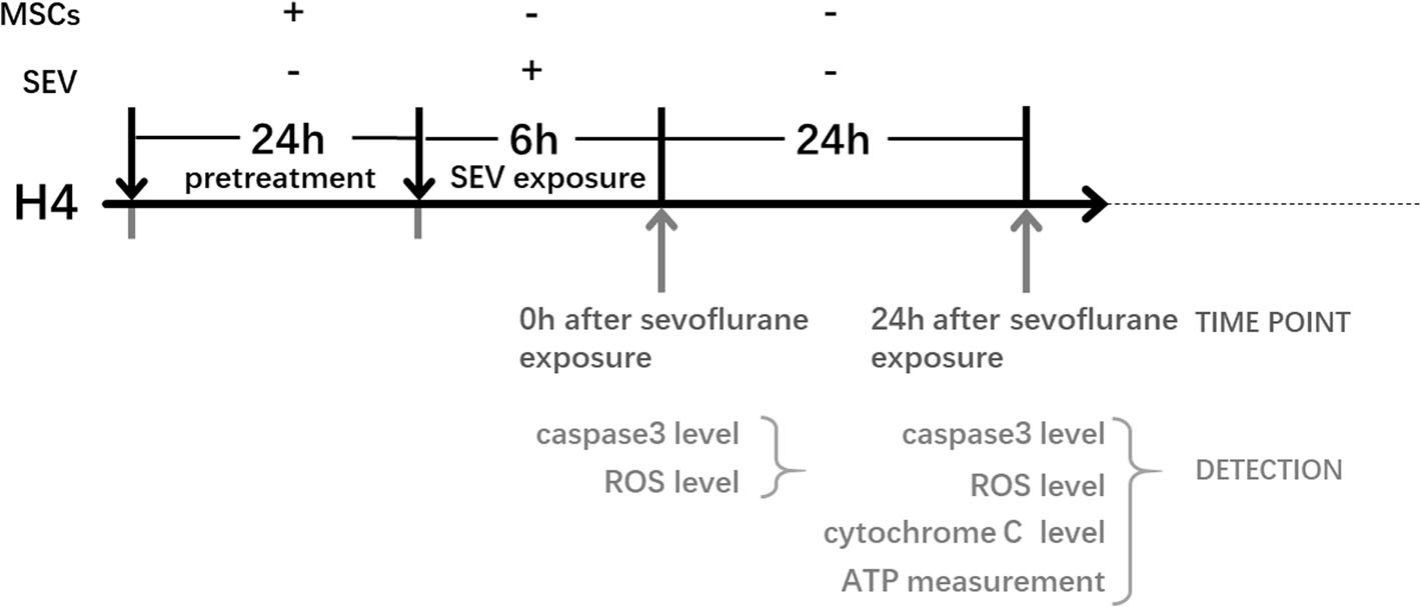
Flowchart of the experimental design

To investigate whether the neurotoxicity of sevoflurane was caused by oxidant stress effect, H4 cells cultured in the presence of oxidants (Rosup, 100 μM, Beyotime) was regarded as a positive control group; Antioxidant N-acetylcysteine (NAC) (1 mM, Beyotime, China) was used to attenuate the sevoflurane-induced ROS level and apoptosis level. The experimental protocol is illustrated in Fig. 1.

### Treatment with MSCs after sevoflurane exposure in H4 cells

H4 cells were cultured alone for 24 h and exposed to 4% sevoflurane for 6 h. Subsequently, the H4 cells were cultured alone or co-cultured with MSCs for 24 h. The levels of intracellular ROS in the different groups of cells were determined by fluorescent microscopy and spectrum.

### Detection of ROS generation

The contents of ROS in H4 cells were determined by fluorescent microscopy and spectrum. Briefly, the harvested H4 cells were treated with 10 mM 2,7-dichlorofluorescein diacetate (DCFH-DA) at 37 °C for 30 min and after being washed, the fluorescent signals in the different groups of H4 cells were observed under a fluorescent microscope (Ti-S, Nikon, Japan). In addition, the contents of fluorescent signals in the H4 cells were determined using a fluorometric microplate reader (Synergy H1, BioTek, USA) at 488 nm.

### Western blotting

The different groups of H4 cells were lyzed in cell lysis buffer. After being centrifuged, the concentrations of total proteins in cell lysates were determined by bicinchoninic acid assay. The cells lysates (30 μg/lane) were separated by sodium dodecyl sulfate polyacrylamide gel electrophoresis on 10% or 12% gels and transferred onto polyvinylidene difluoride membranes. The membranes were blocked with 5% fat-free dry milk in TBST and incubated with primary antibodies against caspase-3 (1:1000 dilution, Cell Signaling Technology #9661, Danvers, MA), Bax (1:1000 dilution, Cell Signaling Technology #2774, Danvers, MA), cytochrome C (1:1000 dilution, Cell Signaling Technology #11940, Danvers, MA), Cox4 (1:1000 dilution, Cell Signaling Technology #4850, Danvers, MA) and β-actin (1:1000 dilution, Cell Signaling Technology #8457, Danvers, MA) at 4 °C overnight. The bound antibodies were detected with horseradish peroxidase-conjugated secondary anti-rabbit IgG (1:1000 dilution, Cell Signaling Technology #7074, Danvers, MA) and visualized using the enhanced chemiluminescence (ECL, Thermo Fisher, USA). The relative levels of target protein to β-actin were determined by densitometric analysis using the Image J software.

In addition, the mitochondrial and cytosol samples were extracted from the different groups of H4 cells using the Cell Mitochondria Isolation Kit (Beyotime, China). The levels of cytochrome C in the mitochondria and cytosol samples were characterized by Western blot using Cox4 and β-actin as the internal references, respectively.

### Adenosine triphosphate (ATP) measurement

The levels of ATP generated by the different groups of cells were determined by bioluminescent assay using the ATP Determination Kit (Invitrogen, USA), according to the manufacturer’s instruction. Briefly, the different groups of H4 cells were cultured in transwell system overnight and exposed to sevoflurane, followed by culturing for another 24 h. The H4 cells were harvested and lyzed in cold lysis buffer (100 μl/well), followed by centrifuging. The lysate samples were reacted in triplicate in 100 μl of the standard reaction solution prepared freshly with reagents provided for 15 min and the luminescence (arbitrary unit) of individual wells was measured at emission of 560 nm in a luminometer (Synergy H1, BioTek, USA). The levels of ATP in individual samples were calculated, according to the standard curve established using different concentrations of ATP provided.

## Statistical analysis

Data were expressed as mean ± SD from each group (*n* = 6 per group). Differences among groups were statistically analyzed by one-way ANOVA and post hoc Fisher’s least significant difference (LSD) using the Prism 5 Software. A *p*-value of less than 0.05 was considered statistically significant.

## Results

### MSCs mitigate H4 cell apoptosis induced by sevoflurane

**Fig. 2:**
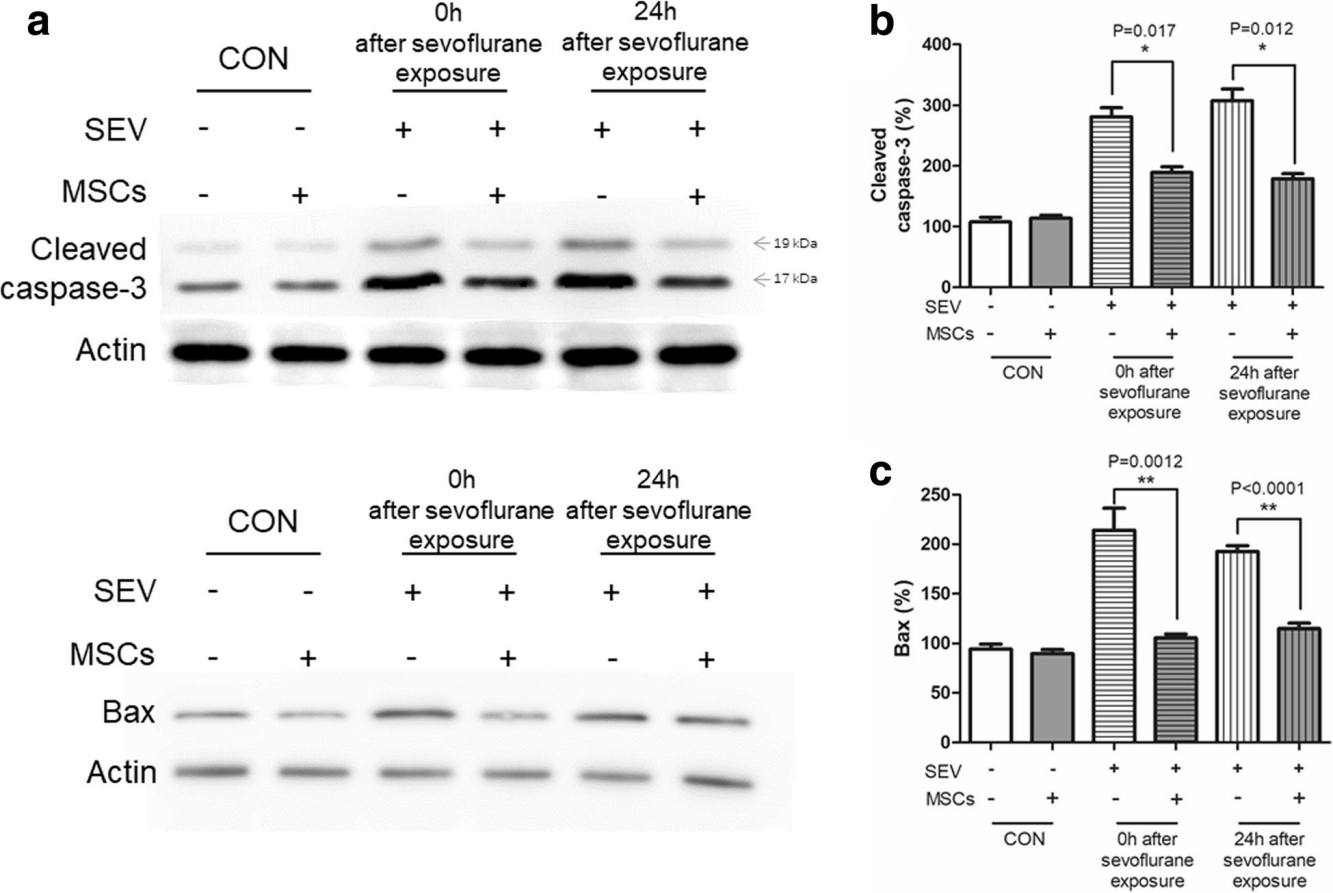
MSCs mitigate the sevoflurane-induced caspase 3 cleavage and Bax expression in H4 cells. H4 cells were cultured alone or co-cultured with MSCs for 24 h and then exposed to 4% sevoflurane for 6 h in transwell plates. Subsequently, some cells from each group were cultured for another 24 h. The relative levels of cleaved caspase 3 and Bax expression in H4 cells were determined after sevoflurane exposure at 0 and 24 h by Western blot. Data are representative images or expressed as the mean ± SD of each group (*n* = 6) from three separate experiments. The levels of each protein expression in the control cells were designated as 100%. **a**. Western blot analysis; (**b**-**c**) Quantitative analysis

We first tested the impact of MSC treatment on the sevoflurane-induced apoptosis in H4 cells by analysis of the relative levels of cleaved caspase 3 (281.2 ± 14.93% and 308.0 ± 18.60% vs. 107.5 ± 7.5% at 0 and 24 h, respectively) and Bax (214.0 ± 12.90% and 214.0 ± 12.91% vs. 94.3 ± 2.84% at 0 and 24 h, respectively) in H4 cells without co-cultured with MSCs (Fig. 2). In contrast, co-cultured of H4 cells with MSCs significantly mitigated the sevoflurane-elevated cleaved caspase 3 (189.1 ± 9.30% vs. 281.2 ± 14.93%, *p* < 0.05; and 179.0 ± 8.49% vs. 308.0 ± 18.60%, p < 0.05) and Bax immediately (0 h, 105.0 ± 2.36% vs. 214.0 ± 12.90%, *p* < 0.01) and 24 h (105.4 ± 2.36% vs. 214.0 ± 12.91%, p < 0.01) after sevoflurane exposure in H4 cells in our experimental system (Fig. 2b and c).

### MSCs attenuate sevoflurane-induced ROS production in H4 cells

**Fig. 3:**
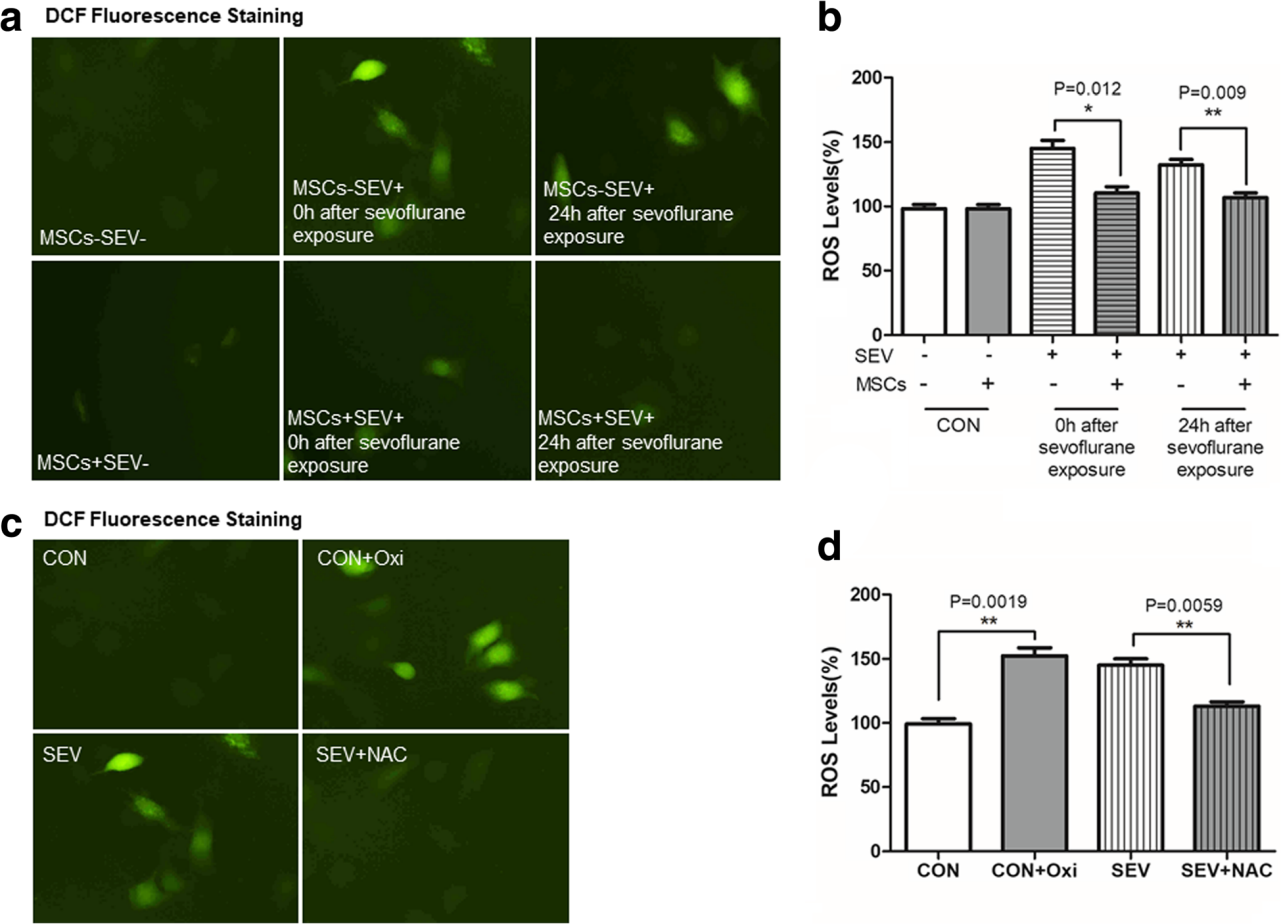
MSCs attenuate the sevoflurane-induced ROS production in H4 cells. The levels of ROS in the different groups of cells were characterized after staining with DCFH-DA by fluorescent microscopy and spectrum. Data are representative images or expressed as the mean ± SD of each group (n = 6) from three separate experiments. **a**. Microscopy characterization of ROS levels in H4 cells. **b**. Quantitative analysis. **c**. NAC scavenges the sevoflurane-induced ROS in H4 cells. H4 cells were treated with, or without, oxidant or exposed to sevoflurane for 6 h in the presence of NAC. The levels of intracellular ROS were measured by fluorescent spectrum. **d**. Quantitative analysis. The levels of ROS in the control cells were designated as 100%

Next, we measured the levels of intracellular ROS in H4 cells by DCFH-DA based fluorescent microscopy and spectrum. First, exposure to sevoflurane significantly increased the levels of cytoplasmic ROS at both immediately (145.0 ± 6.31% vs. 98.2 ± 3.24%) and further 24 h culture (132.4 ± 4.13% vs. 98.2 ± 3.24%) after sevoflurane exposure while co-culture with MSCs almost abrogated the sevoflurane-induced ROS production in H4 cells (110.4 ± 4.90% vs. 145.0 ± 6.31% *p* < 0.05, or 106.9 ± 3.45% vs. 132.4 ± 4.13% *p* < 0.01, Fig. 3a and b). Similarly, treatment with oxidant (152.4 ± 6.07%) induced high levels of ROS production while treatment with NAC (113.1 ± 3.22%) to scavenge ROS dramatically attenuated the sevoflurane-induced ROS production in H4 cells (*p* < 0.01 for all, Fig. 3c and d).

### Sevoflurane-induced apoptosis was attenuated by NAC

**Fig. 4:**
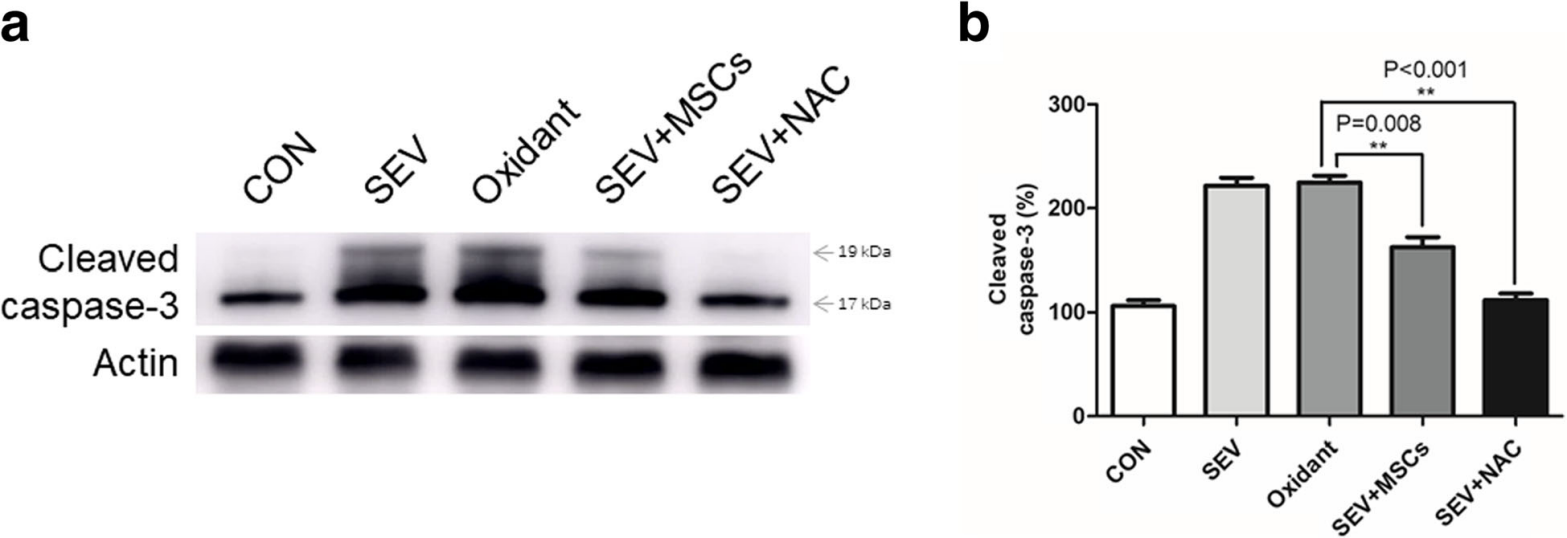
NAC attenuates the sevoflurane-elevated caspase 3 cleavage in H4 cells. H4 cells were treated with vehicle (CON), SEV, Oxidant alone or together with MSCs (SEV + MSCs) or SEV + NAC. The relative levels of cleaved caspase 3 in the different groups of cells were determined by Western blot. Data are representative images or expressed as the mean ± SD of each group (*n* = 6) from three separate experiments. **a**. Western blot analysis. **b**. Quantitative analysis. The levels of each protein expression in the control cells were designated as 100%

We further investigated whether treatment with NAC could modulate the sevoflurane-induced apoptosis of H4 cells by measuring the relative levels of cleaved caspase 3 in the different groups of H4 cells by Western blot. We found that treatment with oxidant, like with sevoflurane (221.7 ± 4.40%), significantly increased the levels of cleaved caspase 3 (224.6 ± 3.92% vs. 106.2 ± 3.17%). However, treatment with MSCs or NAC significantly decreased the relative levels of cleaved caspase 3 in the sevoflurane-exposed H4 cells (162.5 ± 5.64% vs. 224.6 ± 3.92%, *p* = 0.008; 111.9 ± 3.55% vs. 224.6 ± 3.92%, *p* < 0.001 Fig. 4).

#### MSCs reduce the sevoflurane-mediated release of cytochrome C from the mitochondria of H4 cells

**Fig. 5:**
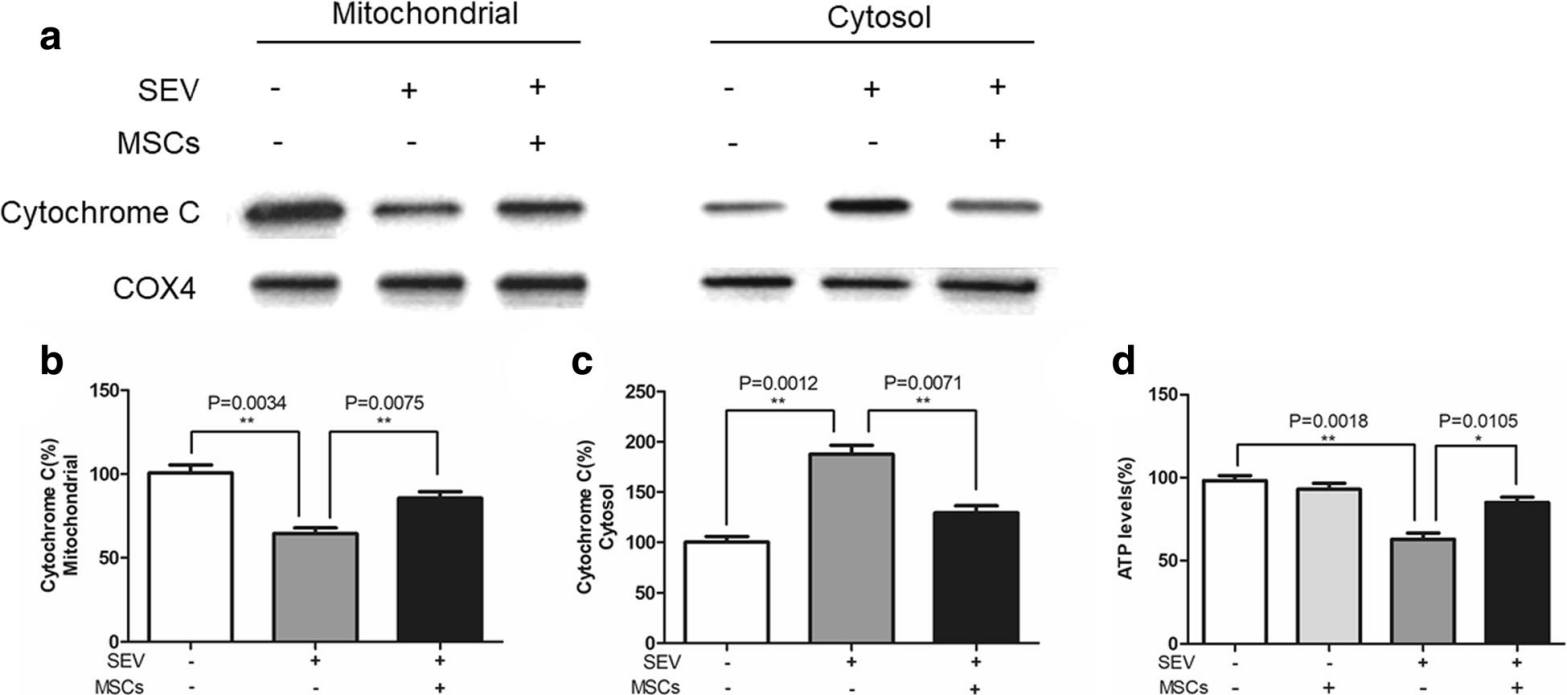
MSCs inhibit the sevoflurane-induced mitochondrial cytochrome C release in H4 cells. The mitochondria and cytosol of the different groups of H4 cells were extracted and the relative levels of cytochrome C were determined by Western blot. Furthermore, the levels of ATP in the different groups of cells were measured. Data are representative images or expressed as the mean ± SD of each group (n = 6) from three separate experiments. **a**. Western blot analysis. **b** and **c**. Quantitative analysis. **d**. The levels of ATP. The levels of each protein expression or ATP in the control cells were designated as 100%

High levels of ROS usually induce cell apoptosis through the mitochondrial pathway. During the process, apoptosis triggers can result in the loss of the mitochondrial transmembrane potential and release of cytochrome C in cells. To understand the action of sevoflurane-mediated apoptosis, we characterized the distribution of cytochrome C and ATP levels in H4 cells. We found that exposure to sevoflurane significantly decreased the levels of mitochondrial cytochrome C (64.47 ± 3.31% vs. 100.7 ± 4.79%, *p* < 0.01), but increased the levels of cytosolic cytochrome C (187.6 ± 9.06% vs. 100.0 ± 5.65%, *p* < 0.01, Fig. 5a-c). In contrast, co-culture with MSCs significantly restored the levels of mitochondrial cytochrome C (85.5 ± 3.95% vs. 64.47 ± 3.31%, *p* < 0.01) and mitigated the sevoflurane-increased cytosolic cytochrome C in H4 cells (129.2 ± 7.08% vs. 187.6 ± 9.06%, *p* < 0.01). Further analysis revealed that sevoflurane exposure significantly reduced the levels of ATP in H4 cells (62.8 ± 3.77% vs. 98.2 ± 2.99%, p < 0.01) while co-culture with MSCs prevented the sevoflurane-decreased ATP levels in H4 cells (85.1 ± 3.14% vs. 62.8 ± 3.77% *p* < 0.05, Fig. 5d).

### MSCs fail to inhibit the sevoflurane-induced ROS production in H4 cells post sevoflurane exposure

**Fig. 6:**
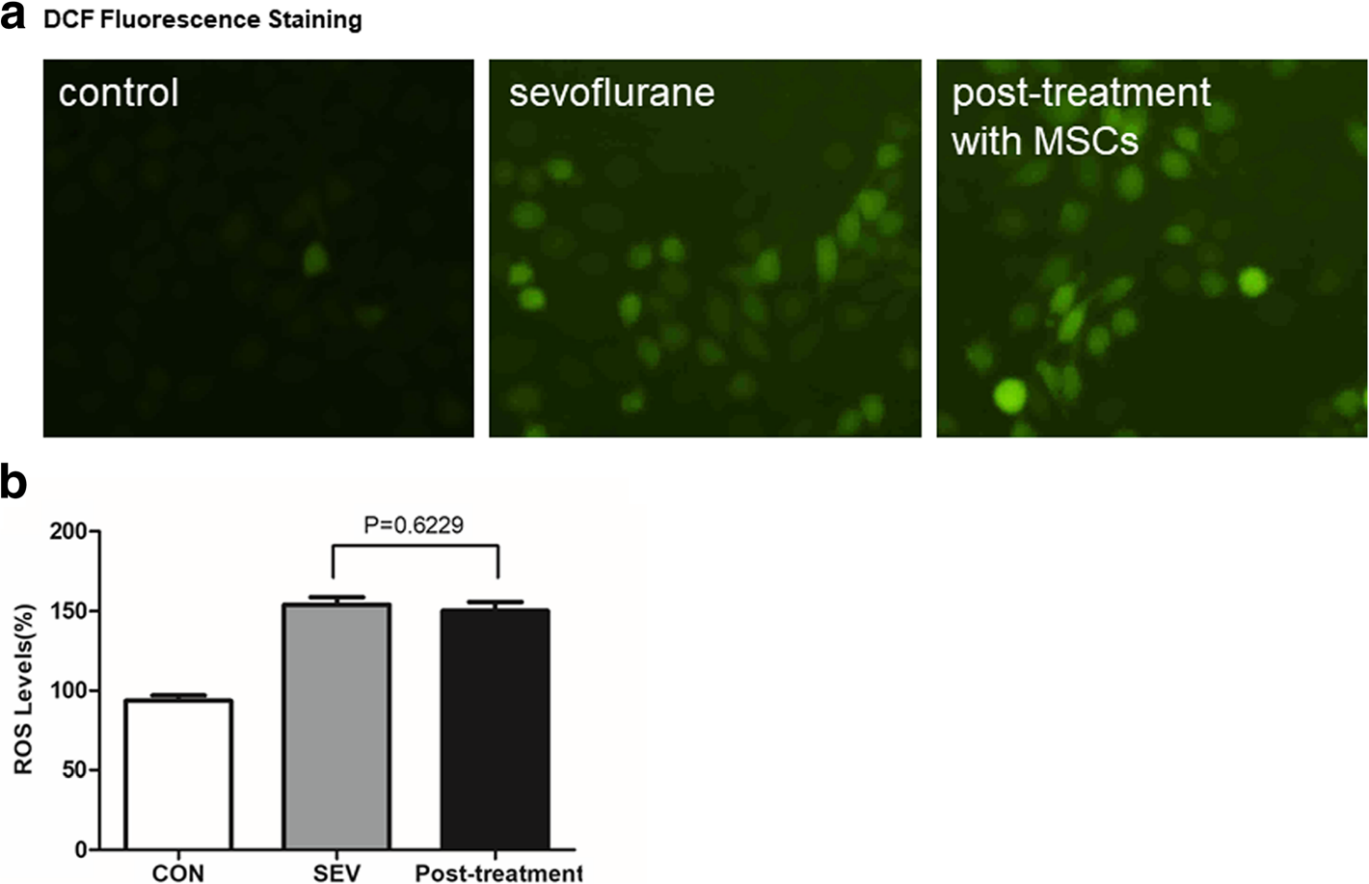
Co-culture with MSCs after sevoflurane exposure does not alter the sevoflurane-induced ROS production in H4 cells. H4 cells were cultured for 24 h and exposed to sevoflurane, followed by co-cultured with MSCs or cultured alone for 24 h. The control H4 cells were cultured alone throughout the experimental period. The cells were stained with DCFH-DA and the levels of ROS were determined by fluorescent microscopy and spectrum. Data are representative images or expressed as the mean ± SD of each group (n = 6) from three separate experiments. **a**. Fluorescent microscopy analysis of ROS in H4 cells. **b**. Fluorescent spectrum analysis of the ROS levels in H4 cells. The levels of ROS in the control cells were designated as 100%

Finally, we tested whether treatment with MSCs post exposure to sevoflurane could inhibit the sevoflurane-induced ROS production in H4 cells. The levels of ROS were 93.7 ± 3.28% (CON), 154.0 ± 4.69% (SEV), 150.2 ± 5.55% (Post-treatment), respectively. We found that sevoflurane exposure significantly induced high levels of ROS production (154.0 ± 4.69% vs. 93.7 ± 3.28%) and co-culture with MSCs after sevoflurane exposure did not change the levels of sevoflurane-induced ROS production in H4 cells (150.2 ± 5.55% vs. 154.0 ± 4.69%, *p* > 0.05, Fig. 6).

## Discussion

In this study, we found that treatment with MSCs before, but not after, sevoflurane exposure attenuated the sevoflurane-induced ROS production and apoptosis in H4 cells, which was abrogated by antioxidant NAC. MSCs prevented the sevoflurane-induced cytochrome C release from the mitochondria to the cytoplasm and ATP production in H4 cells. These novel data extended previous observations [17–22] and indicated that soluble factors secreted by MSCs had potent antioxidant activity against oxidative stress-induced apoptosis in H4 cells. These finding may provide new insights into the neuronal toxicity of sevoflurane. Given that sevoflurane is an inhaled anesthetic used widely in the clinical practice in anesthesia our findings suggest that we should be cautious while using sevoflurane.

MSCs can secrete neurotrophic factors, cytokines and other soluble factors that are associated neuroprotective activity [17–20]. In this study, while sevoflurane exposure induced ROS production, mitochondrial cytochrome C release and apoptosis in H4 cells co-culture with MSCs before sevoflurane exposure almost completely prevented the oxidant activity of sevoflurane in H4 cells. However, we found that co-culture with MSCs after sevoflurane exposure failed to mitigate the sevoflurane-induced ROS production in H4 cells. These data suggest that sevoflurane may trigger an oxidative cascade that induces the mitochondrial damages and apoptosis of H4 cells, which may not be easily overcome by soluble factors from MSCs [29]. A recent study has shown that infusion of MSCs improves symptoms in patients with Alzheimer’s disease [30]. Further studies are necessary to identify soluble factors secreted by MSCs and determine the molecular mechanisms underlying the action of MSCs.

Given that this experiment was performed in transwell plates the antioxidant effect of MSCs was likely mediated by their soluble factors. Indeed, MSCs can secrete neurotrophic factors, cytokines and other extracellular vesicles (EVs). Our findings support the theory that the EVs secreted by MSCs are responsible for their immunosuppressive activity [31] and the neuronal protection by MSCs may be independent of cell-to-cell contact. It is notable that EVs include exosomes, ectosomes, microvesicles, microparticles, apoptotic bodies and other EV subsets [32]. We are interested in further investigating which type of EV(s) has such potent antioxidant activity and neuronal protective effect. Given that most EVs are able to pass through the blood-brain barrier and are relatively stable the identified EVs or soluble factors may be valuable for cell-free therapy of oxidative stress-related neuronal degenerative diseases.

We recognized that our study has limitations. First, the MSCs were extracted from newborn SD rats and tested in a human cell line. However, MSCs have the advantage of low-immunogenicity which make them potentially safe for clinical use. Because of MSC’s immunomodulatory effects, they can be used to repair the damage caused by autoimmune-induced disease and graft-versus-host disease [33]. Autologous and allogenic bone marrow derived MSCs have been shown to be safe for human use [34, 35]. Xenogeneic bone marrow derived MSCs from human, mouse and rat have been widely used in animal experiments [36–38]. Because of the role of MSC-derived EVs, EVs isolated from conditioned media of MSCs would be used in our further animal studies. Second, although we used an optimal dose of MSCs in our experiments we did not test the time course of the co-culture system in detail. Once MSCs and H4 cells were seeded into their own chambers respectively, the co-culture system was started. After culture for 24 h, the cells should be in the best condition of density and the sevoflurane exposure started. Further studies are needed to test whether co-culture for varying time periods or culture for a longer period after exposure to sevoflurane has different effects on sevoflurane-induced apoptosis and ROS production because co-culture for 24 h or exposure to sevoflurane for 6 h may be not optimal. Third, these results were based on cell experiments and still need to be confirmed in animals.

## Conclusions

Sevoflurane is the most widely used inhaled anesthetic in clinical practice. In this study, we demonstrated that the exposure to 4% sevoflurane for 6 h induced ROS production, apoptosis and caspase-3 activation in H4 cells. Furthermore, we revealed that the sevoflurane-mediated apoptosis was mediated by the mitochondrial pathway. In addition, we found for the first time that the co-culture system with MSCs reduced the toxic effect of sevoflurane on H4 cells. Potentially, our findings may aid in design of new therapies for prevention of sevoflurane-induced neuronal damage.

## Abbreviations

ATP: Adenosine triphosphate
DCFH-DA: 2,7-dichlorofluorescein diacetate
DMEM: Dulbecco’s modified eagle medium
FBS: Fetal bovine serum
H4 cells: Human neuroglioma H4 cells
MSCs: Mesenchymal stem cells
NAC: N-acetylcysteine
ROS: Reactive oxygen species
SD rats: Sprague-Dawley rats
